# Review of Prof. Noel's Article on Dentine

**Published:** 1870-06

**Authors:** 


*Review of Prof. Noels Article on Dentine.*—Prof. Judd in the April No. of the *Missouri Dental Journal*, reviews this article, which appeared in the Feb. No. of the *American Journal of Dental Science*, as follows: "Commencing at the completion of the sacular stage of development, Prof Noel describes the secondary histogenetic process very clearly, but differs, in some particulars, from the doctrines laid down by Tomes and Kölliker, following, for the most part, the peculiar language and description of Beale. Although describing the histogenetic process somewhat differently from the latter author, he arrives at the same conclusions so far as the most important points are concerned. The first point of divergence from the commonly accepted doctrine is found in the statement that masses of germinal matter are found upon the external surface of the pulp, i. e., external to, and resting upon the tunica propria, which extend themselves toward the masses that proceed from the tunica reflexa; that these meet, and that the one forms the enamel organ and the the other the dentinal organ, which, when calcified, form enamel and dentine. That a dentinal organ is formed external to the tunica propria will be new to many who have been accustomed to regard the process of dentinification as being carried on through the action of the cellular element which forms the periphery of the papilla or pulp proper.

According to the theory here put forth, the essential difference between enamel and dentine consists in the degree to which conversion of germinal matter to formed material has taken place, that while all of the germinal matter has been converted into formed material in the enamel, the central portion of the tubuli, or as he calls them, fibrillæ, still remains germinal matter.

Conclusion ninth to which he arrives reads—Fresh dentine, therefore, has no tubuli, but has uncalcified fibrillæ solid, or nearly solid.

We must confess that we do not exactly understand why a set of hollow rods should be converted into fibrillæ, and declared not to be tubuli, because they are filled with a fluid or soft solid matter. Fill a hard tube with water, or with any other substance that does not blend with it and make a part of the solid substance itself, and it will not change the tube into a fibre, or in any way destroy its identity.

The twelfth conclusion, which expresses the opinions of Beale, is not in accordance with views generally held upon the subject. " Enamel and dentine are not vitalized tissues so far as the calcified portions are concerned. "Enamel is dead—perfectly dead, &c.," does not exactly agree with the opinions one could naturally form from a careful consideration of a few points which are laid down by the author himself. When speaking of the enamel organ, he remarks, "this is the enamel's organic base." Now, if we understand by "organic base" vitalized tissue, would we expect that this vitalized base would be *perfectly dead* as soon as some phosphate of lime and a few other salts were deposited among its elements, and is it moreover probable that this base of connective tissue, if it were dead, would remain unchanging and unchanged during a long term of years, forming not only an exception to the general law which dominates over all other soft tissues, but also militating against that high law which ordains that constant change is stamped upon every atom of matter throughout the universe?

If we were asked to furnish evidence that enamel was not dead—perfectly dead—we would point to the fact, that it is many times exceedingly sensitive to the touch, and that sensibility is one of the most certain signs of vitality wherever it exists. Can any one give as good a reason for supposing it to be dead?"

